# Supplementary material for: Dose-Response Relationships between Breastfeeding and Postpartum Weight Retention Differ by Pre-Pregnancy Body-Mass Index in Taiwanese Women
Source: Nutrients. 2020 Apr 11;12(4):1065. doi: 10.3390/nu12041065 (PMC7231130; doi:10.3390/nu12041065)
Supplement: Supplementary file 1 [file nutrients-12-01065-s001.pdf]

Supplementary materials

Table S1. Adjusted means and 95% confidence intervals of postpartum weight retention for exclusive, partial and not breastfeeding groups by breastfeeding duration (N= 52,367).

	exclusive breastfeeding				partial breastfeeding				not breastfeeding			
**breastfeeding duration, days**	**n**	**mean, kg**	**lower 95% CI**	**higher 95% CI**	**n**	**mean, kg**	**lower 95% CI**	**higher 95% CI**	**n**	**mean, kg**	**lower 95% CI**	**higher 95% CI**
0	13791	2.91	2.85	2.98	223	2.91	2.81	3.02	n/a	n/a	n/a	n/a
30	4056	2.69	2.64	2.74	5163	2.91	2.83	2.98	2339	2.97	2.85	3.08
60	2649	2.49	2.43	2.55	3629	2.88	2.82	2.94	1473	2.99	2.88	3.10
90	2265	2.30	2.24	2.37	2763	2.83	2.78	2.88	1008	3.01	2.88	3.15
120	6457	2.13	2.07	2.19	2805	2.76	2.70	2.81	957	3.03	2.88	3.19
150	4026	1.98	1.92	2.03	1478	2.66	2.61	2.72	356	3.06	2.90	3.21
180	8576	1.84	1.77	1.90	4090	2.55	2.49	2.60	1061	3.08	2.89	3.27

n/a – not available.

Table S2. Adjusted means and 95% confidence intervals of postpartum weight retention for exclusive, partial and not breastfeeding groups by breastfeeding duration and pre-pregnancy body-mass (BMI) index (N= 52,367).

**BMI**	exclusive breastfeeding				partial breastfeeding				not breastfeeding			
**underweight, lower than 18.5**	**n**	**mean, kg**	**lower 95% CI**	**higher 95% CI**	**n**	**mean , kg**	**lower 95% CI**	**higher 95% CI**	**n**	**mean, kg**	**lower 95% CI**	**higher 95% CI**
breastfeeding duration, days												
0	2,272	2.75	2.62	2.89	33	2.73	2.53	2.94	n/a	n/a	n/a	n/a
30	842	2.61	2.51	2.71	1006	2.73	2.59	2.88	356	2.92	2.70	3.14
60	491	2.47	2.36	2.58	657	2.72	2.61	2.83	214	2.94	2.74	3.15
90	396	2.35	2.22	2.47	513	2.69	2.59	2.79	187	2.96	2.71	3.21
120	1,076	2.23	2.11	2.35	453	2.64	2.54	2.75	91	2.97	2.69	3.24
150	665	2.13	2.02	2.23	282	2.58	2.47	2.69	199	2.97	2.68	3.26
180	1,425	2.03	1.91	2.16	671	2.50	2.39	2.62	66	2.97	2.62	3.32
normal, 18.5-23.9												

breastfeeding duration, days												
0	9,052	2.96	2.89	3.04	144	2.98	2.85	3.11	n/a	n/a	n/a	n/a
30	2,556	2.74	2.68	2.80	3220	2.98	2.88	3.07	1,251	3.01	2.86	3.16
60	1,750	2.54	2.47	2.61	2384	2.95	2.88	3.02	755	2.99	2.86	3.12
90	1,495	2.35	2.28	2.43	1760	2.89	2.83	2.96	732	3.01	2.84	3.17
120	4,449	2.18	2.11	2.26	1929	2.82	2.76	2.88	295	3.04	2.86	3.22
150	2,793	2.03	1.97	2.10	972	2.72	2.65	2.79	769	3.10	2.91	3.29
180	5,818	1.90	1.82	1.98	2781	2.60	2.53	2.67	214	3.19	2.96	3.42
overweight, 24-26.9												
breastfeeding duration, days												
0	1,520	3.04	2.82	3.27	23	3.28	2.92	3.64	n/a	n/a	n/a	n/a
30	399	2.78	2.60	2.96	557	3.18	2.92	3.44	202	3.23	2.83	3.63
60	253	2.53	2.32	2.74	374	3.06	2.86	3.27	131	3.22	2.80	3.64
90	231	2.30	2.06	2.53	314	2.93	2.74	3.13	93	3.21	2.68	3.75
120	609	2.07	1.84	2.30	262	2.79	2.58	3.00	55	3.23	2.64	3.81
150	388	1.85	1.64	2.06	140	2.63	2.41	2.86	115	3.25	2.65	3.85
180	897	1.65	1.41	1.88	411	2.46	2.24	2.69	33	3.29	2.56	4.01
obese, 27 or higher												
breastfeeding duration, days												
0	947	2.61	2.31	2.92	23	2.26	1.80	2.72	n/a	n/a	n/a	n/a
30	259	2.26	2.01	2.51	380	2.38	2.05	2.72	116	2.38	1.85	2.92
60	155	1.92	1.62	2.22	214	2.45	2.18	2.71	73	2.63	2.13	3.12
90	143	1.59	1.25	1.92	176	2.45	2.19	2.72	47	2.70	2.08	3.31
120	323	1.26	0.94	1.59	161	2.40	2.11	2.68	33	2.59	1.88	3.31
150	180	0.95	0.66	1.24	84	2.28	1.97	2.59	52	2.32	1.53	3.10
180	436	0.64	0.31	0.98	227	2.11	1.79	2.42	30	1.87	0.97	2.77

Table S3. Adjusted means and 95% confidence intervals postpartum weight retention for partial breastfeeding by pre-pregnancy body-mass index (N= 52,367).

**underweight, lower than 18.5**	**n**	**mean, kg**	**lower 95% CI**	**higher 95% CI**
breastfeeding duration, days				
0	33	2.73	2.53	2.94
30	1006	2.73	2.59	2.88
60	657	2.72	2.61	2.83
90	513	2.69	2.59	2.79
120	453	2.64	2.54	2.75
150	282	2.58	2.47	2.69
180	671	2.50	2.39	2.62
210	281	2.41	2.30	2.52
240	276	2.30	2.19	2.41
270	123	2.18	2.07	2.28
300	108	2.04	1.92	2.15
330	43	1.88	1.73	2.03
360	24	1.71	1.51	1.91
normal, 18.5-23.9				
breastfeeding duration, days				
0	144	2.98	2.85	3.11
30	3220	2.98	2.88	3.07
60	2384	2.95	2.88	3.02
90	1760	2.89	2.83	2.96
120	1929	2.82	2.76	2.88
150	972	2.72	2.65	2.79
180	2781	2.60	2.53	2.67
210	1225	2.46	2.39	2.53
240	1189	2.30	2.23	2.36
270	548	2.11	2.05	2.17
300	458	1.90	1.83	1.97
330	223	1.67	1.58	1.76
360	90	1.42	1.29	1.54
overweight, 24-26.9				
breastfeeding duration, days				
0	23	3.28	2.92	3.64
30	557	3.18	2.92	3.44
60	374	3.06	2.86	3.27

90	314	2.93	2.74	3.13
120	262	2.79	2.58	3.00
150	140	2.63	2.41	2.86
180	411	2.46	2.24	2.69
210	179	2.28	2.06	2.50
240	158	2.08	1.88	2.29
270	82	1.87	1.68	2.06
300	62	1.65	1.43	1.86
330	24	1.41	1.12	1.69
360	14	1.15	0.76	1.55
obese, 27 and higher				
breastfeeding duration, days				
0	23	2.26	1.80	2.72
30	380	2.38	2.05	2.72
60	214	2.45	2.18	2.71
90	176	2.45	2.19	2.72
120	161	2.40	2.11	2.68
150	84	2.28	1.97	2.59
180	227	2.11	1.79	2.42
210	104	1.87	1.56	2.18
240	84	1.58	1.29	1.87
270	43	1.22	0.94	1.50
300	34	0.81	0.50	1.11
330	10	0.33	-0.06	0.73
360	3	-0.20	-0.74	0.34

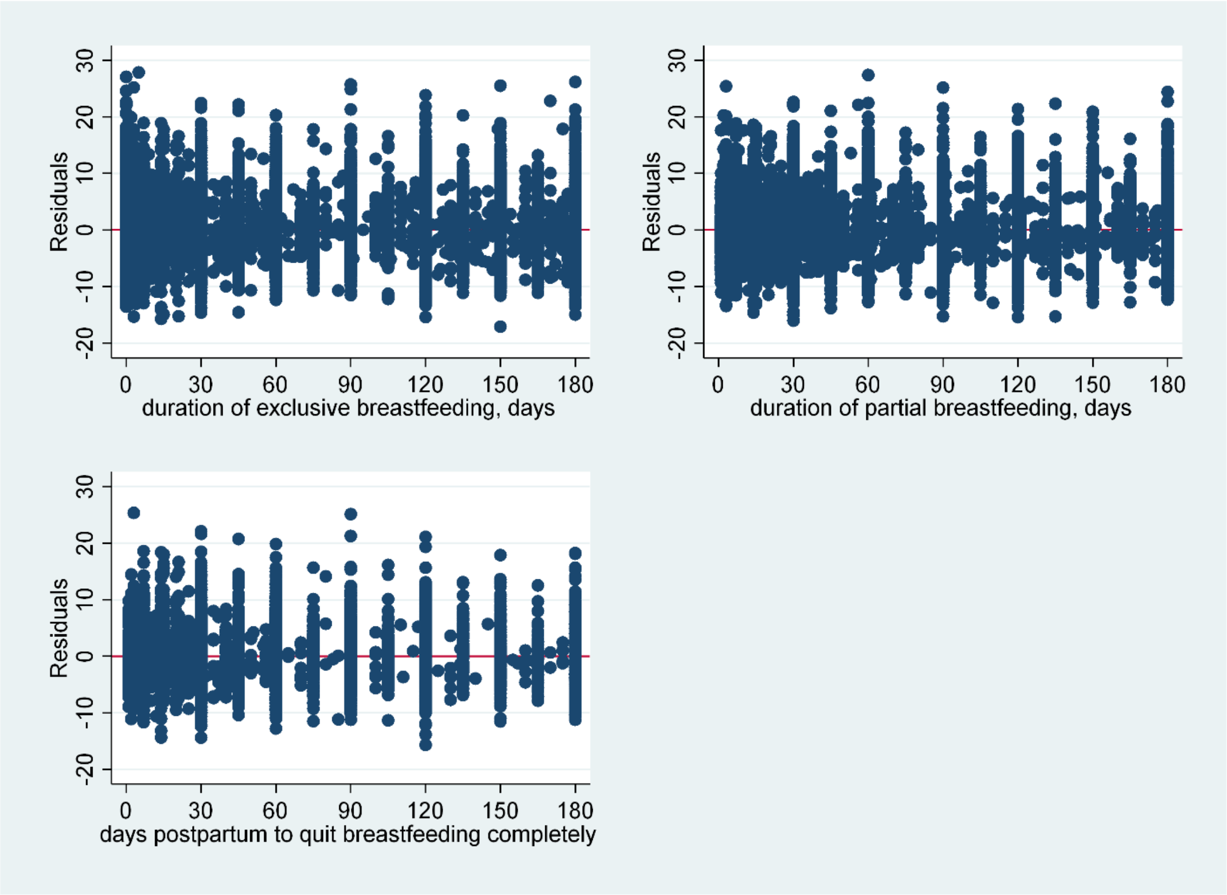

Figure S1. Residuals plots for models in figure 1.

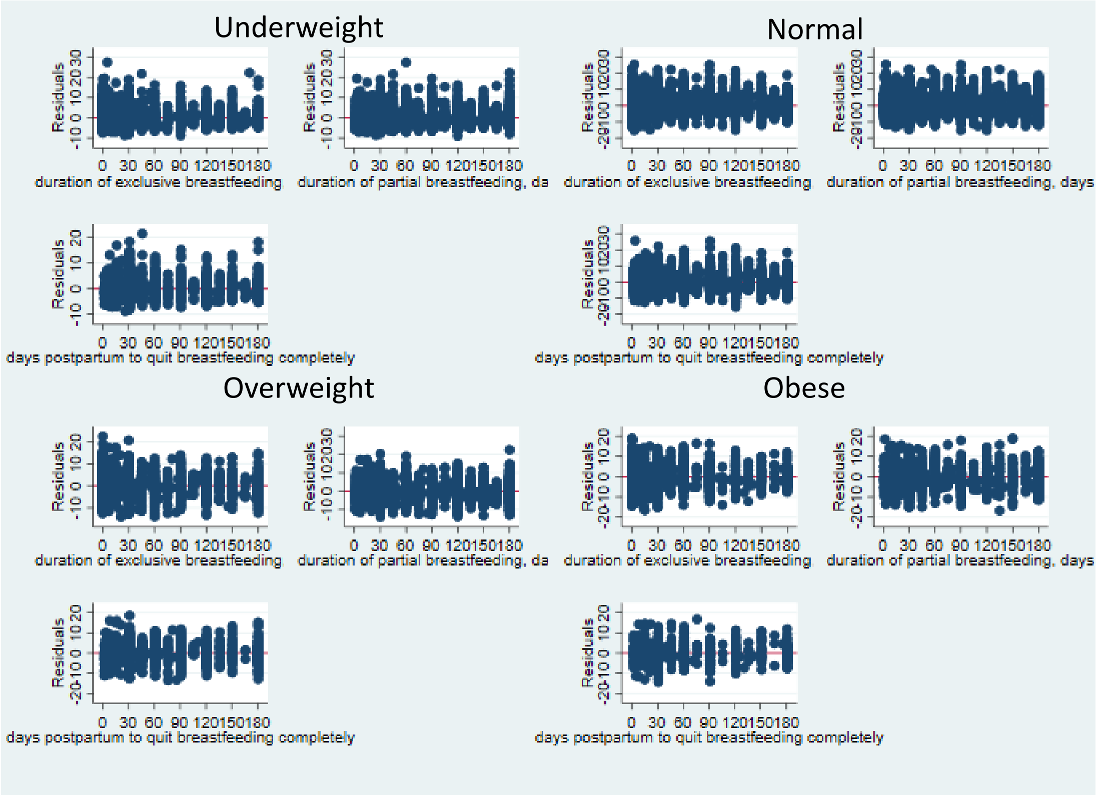

Figure S2. Residuals plots for models in figure 2.

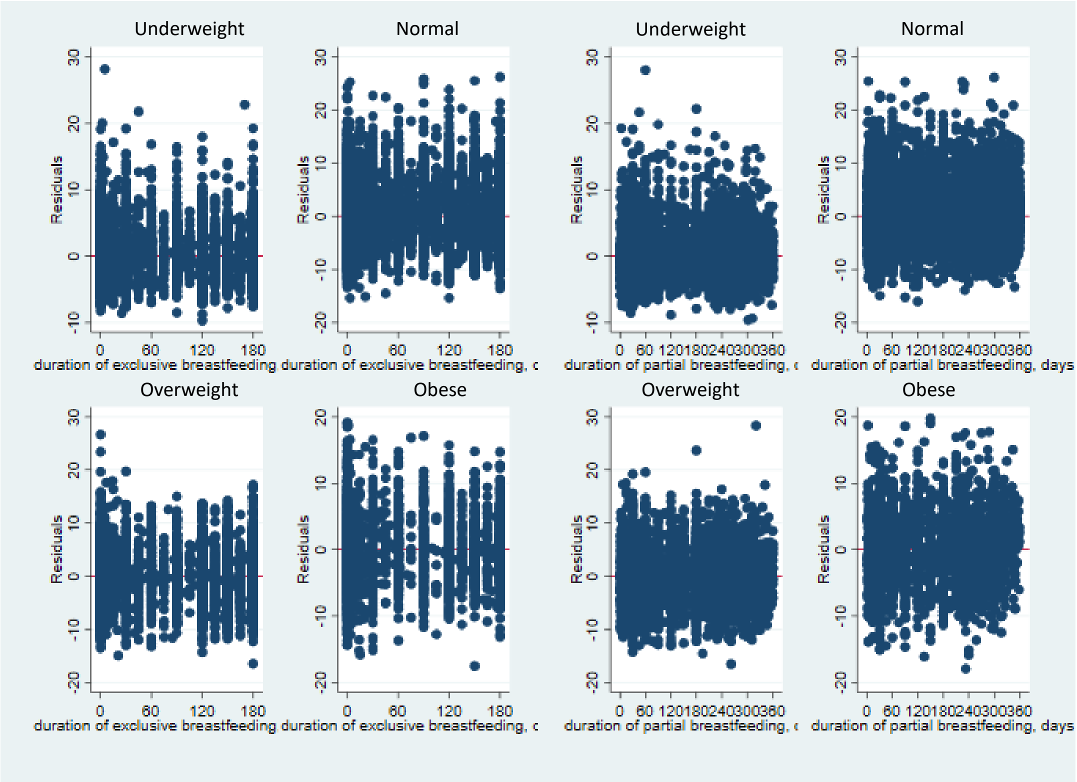

Figure 3. Residuals plots for models in figure 3.

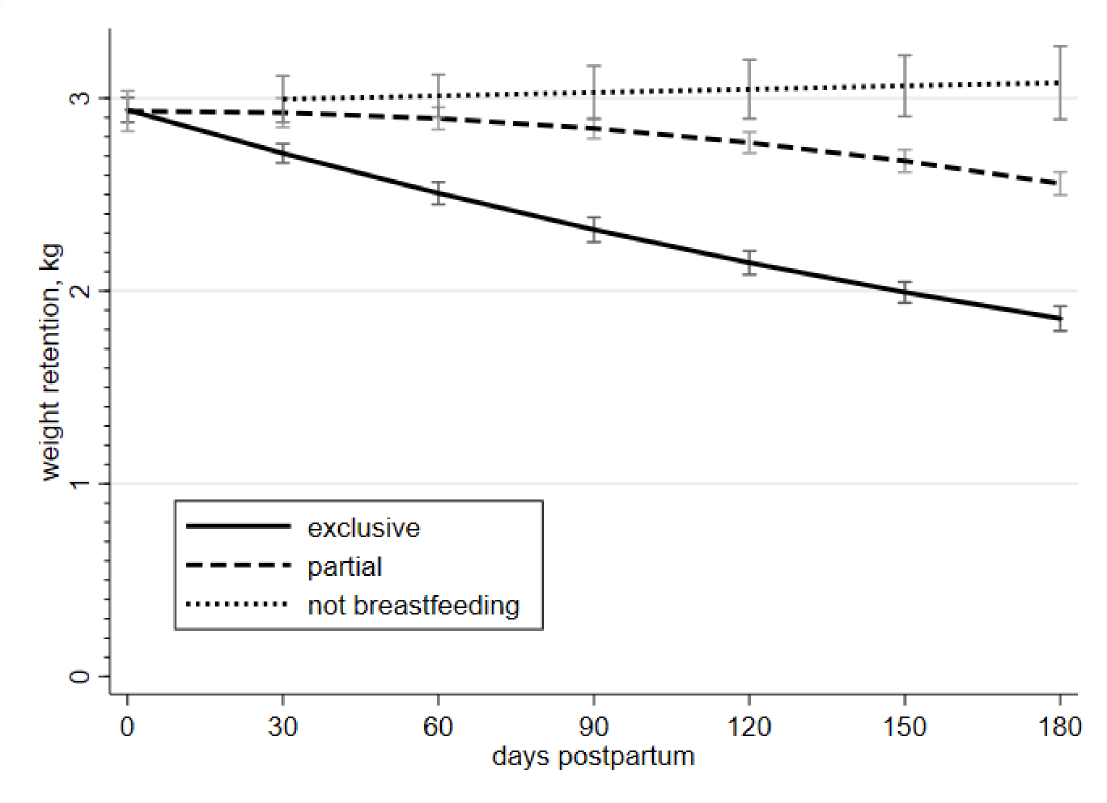

Figure S4. Adjusted means and 95% confidence intervals of postpartum weight retention and duration of breastfeeding in Taiwanese women with singletons (N = 50,994).

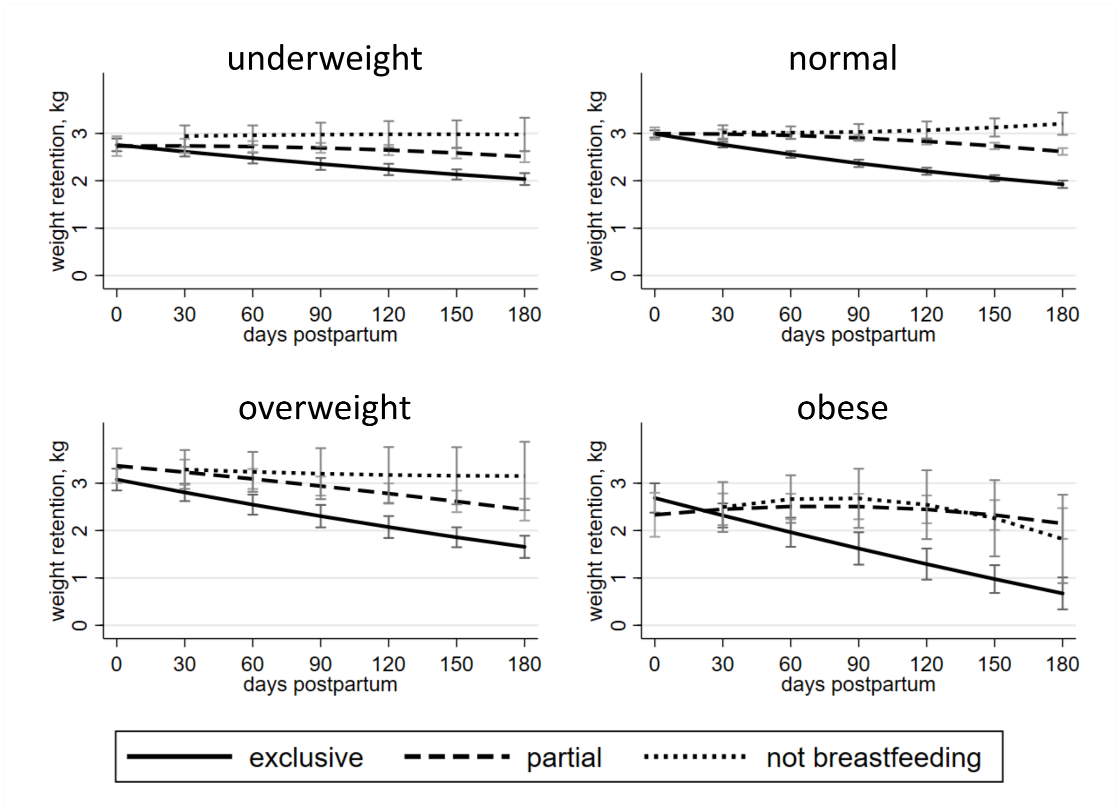

Figure S5. Adjusted means and 95% confidence intervals of postpartum weight retention and duration of breastfeeding in Taiwanese women with singletons by pre-pregnancy BMI (N = 50,994).

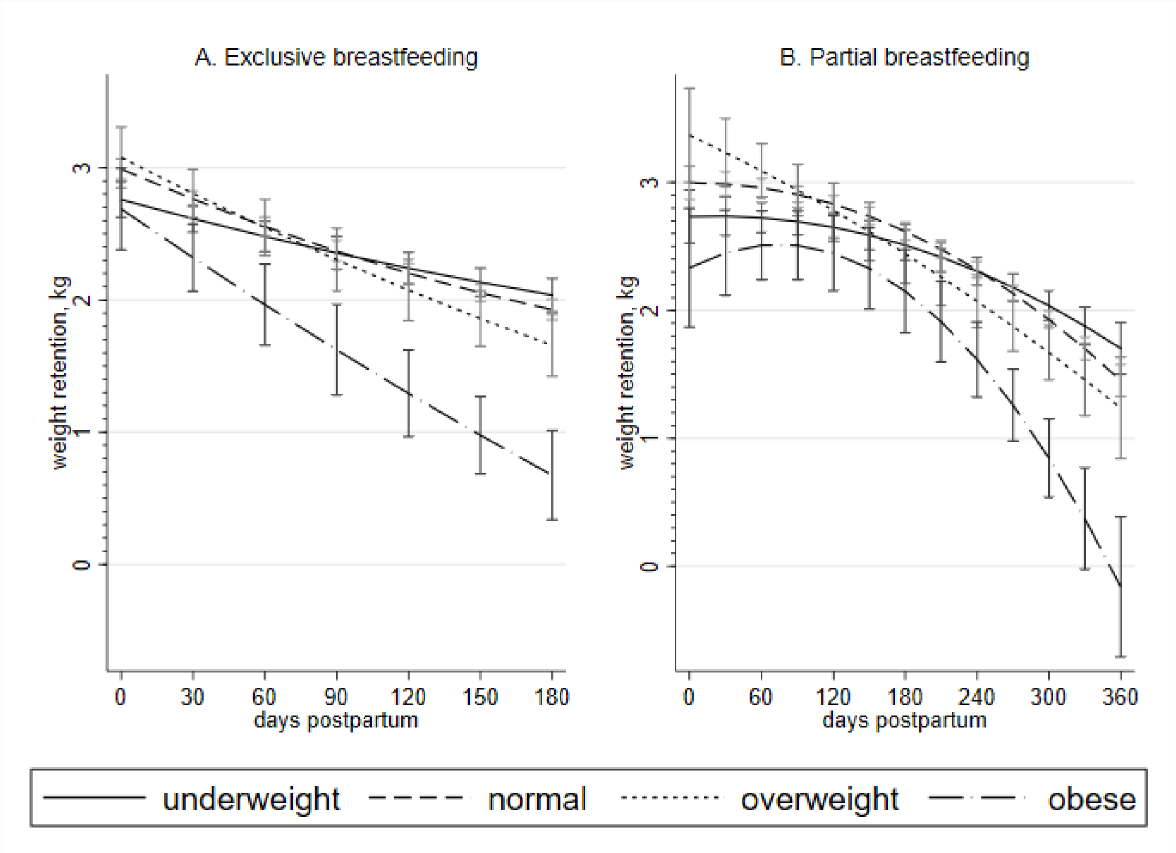

Figure S6. Postpartum weight retention and duration of breastfeeding in Taiwanese women with singletons by pre-pregnancy BMI (N = 50,994).
